# Treatment options for patients with triple-negative breast cancer

**DOI:** 10.1186/1756-8722-3-42

**Published:** 2010-10-27

**Authors:** Rafael Santana-Davila, Edith A Perez

**Affiliations:** 1 Division of Neoplastic Diseases and Related Disorders Medical College of Wisconsin, 9200 W. Wisconsin Ave, Milwaukee, WI 53226 USA; 2 Division of Hematology and Oncology Mayo Clinic, 4500 San Pablo Road, Jacksonville, Florida. 32224. USA

## Abstract

Breast cancer is a heterogeneous disease composed of different subtypes, characterized by their different clinicopathological characteristics, prognoses and responses to treatment. In the past decade, significant advances have been made in the treatment of breast cancer sensitive to hormonal treatments, as well as in patients whose malignant cells overexpress or amplify HER2. In contrast, mainly due to the lack of molecular targets, little progress has been made in the treatment of patients with triple-negative breast cancer. Recent improved understanding of the natural history, pathophysiology, and molecular features of triple-negative breast cancers have provided new insights into management and therapeutic strategies for women affected with this entity. Ongoing and planned translational clinical trials are likely to optimize and improve treatment of women with this disease.

## Introduction

Breast cancer affected an estimated 192,370 women and men in 2009, and was responsible for 40,170 deaths during the same year [1]. It is now clear that it is a disease composed of multiple subgroups characterized by their pathophysiological features, outcomes, and responses to treatment. The heterogeneity of this disease underscores the need for treatments to be tailored for a specific patient, depending on the molecular characteristics of their malignancy.

An initial subdivision of patients with breast cancer can be done by immunohistochemical techniques separating those whose malignant cells express either estrogen (ER) or progesterone receptors (PgR) and those that do not, as the first two can be treated with endocrine therapy. Immunohistochemistry (IHC) or fluorescence in situ hybridization (FISH) can also detect the overexpression (or amplification) of the human epidermal growth factor receptor 2 (HER2), which can also be targeted therapeutically with antibodies or small molecule tyrosine kinase inhibitors. Tumors that do not express ER, PgR, or HER2 are commonly referred to as triple-negative breast cancer (TNBC).

Further understanding of the biology of breast cancer comes from studies that have identified gene expression profiles that provide insight into therapeutic strategies, although more work remains to be done [2-6]. Perou and colleagues [4,5] proposed an initial classification in which breast cancer was subdivided into four groups: Luminal types A and B, HER2 positive cancer and basal-like subset. Luminal type A is characterized by neoplasms that express ER and have a low-grade histology. Luminal type B is composed mostly of tumors with low ER expression and a higher grade compared to those with type A. HER2 positive cancers are distinguished by the amplification of the HER2 gene. Finally, the basal-like subset, which is composed mostly of ER and HER2 negative cancers. This is, of course, an oversimplification of the heterogeneity of breast cancer, albeit helpful based on the current status of knowledge.

## TNBC and Basal-like Cancer

**Figure 1:**
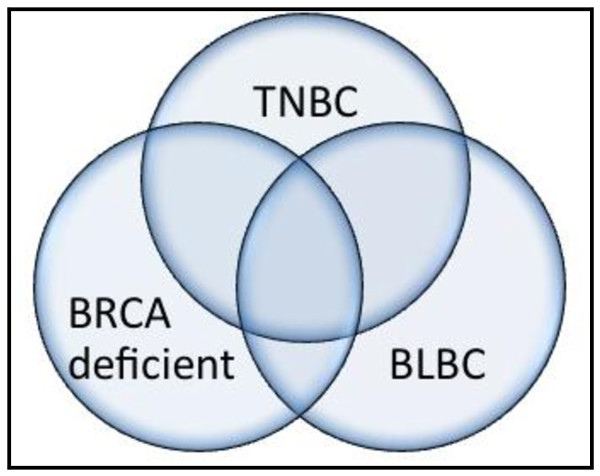
**Schematic diagram the represents the significant overlap that exists between triple-negative (TNBC), basal-like breast cancer (BLBC) and breast cancer that arises in patients who have a BRCA mutation**. While the majority of cancers that are TNBC are also BLBC. Non-basal triple-negative breast cancer also exists. Similarly most breast cancers that occur in women with BRCA mutations are TNBC and of the BLBC subtype, however this overlap is not complete.

Although the terms TNBC and basal-like cancer are often used interchangeably, it is important to clarify that not all TNBCs belong to the basal-like subtype (Figure 1). Although one of the key features of most basal-like cancers is the low expression of hormonal receptors and HER2 related genes, they are also characterized by other features. This was illustrated in the study by Parker and collaborators who, in an attempt to incorporate gene expression based "intrinsic" molecular subtypes for prognosis and prediction of chemotherapy benefit, applied a 50 gene expression signature (PAM50) to a cohort of 1,004 patients, of which 626 had ER positive disease. In this group the majority (73%) were luminal (A or B), but 11% were HER2-enriched, 5% were basal-like, and 12% were normal-like [7]. Similarly, in the ER negative group, 11% of the tumors were found to be luminal, 32% HER2-enriched, 50% basal-like, and 7% normal-like. Their work, and that of others, demonstrated that ER and HER2 status is not an accurate surrogate for true intrinsic subtype status (differentiation between luminal A, luminal B, HER2 and basal-like) [8].

As we wait for validation and further research related to several proposed gene profiles, several investigators have used expression of basal/myoepithelial cell proteins identified by immunohistochemical staining, as a surrogate of gene expression [9-11]. The most widely used panel is based on the expression of cytokeratin 5/6 (CK5/6) and/or the epidermal growth factor receptor (EGFR) in tumors that are triple-negative [12]; however, no uniform consensus exists as to what is the optimal immunnohistochemical panel to identify basal-like breast cancer. Thus TNBC, despite having an imperfect correlation [9,13,14], is generally used clinically as a marker of being a basal-like cancer.

## Rationale for the Term Basal-like Breast Cancer

The normal human breast ducts and acini are composed of two cell layers, which include an inner luminal cell population and a distinct outer cell layer juxtaposed to the basement membrane, named the myoepithelial or basal layer. Cells from each layer have a distinct immunophenotypic profile. Basal-like cancer cells commonly express some of the basal cell markers such as cytokeratin 5 (CK5) and 17 (CK17), as well as caveolin-1, EGFR, B-crystallin, P-cadherin, and c-*KIT *[15-17]. This does not necessarily imply that basal-like tumors arise from the myoepithelial layer; this area remains the focus of intensive investigation[18].

## Clinicopathological Characteristics

Approximately 15-20% of breast cancers are TNBC [19,20], the majority of which are from the basal-like subtype. Basal-like cancers are typically associated with a higher histological grade, marked cellular pleomorphism, a high Ki67 index, increase mitotic activity and atypical mitotic figures[9,21-24]. At the genomic level, in comparison with other subtypes, the basal-like subtype is distinguished by genomic instability, an increase in DNA copy number changes, and frequent low-level gains and deletions [25,26]. This subtype is also characterized by deregulation of important components of the cell cycle process, such as the *RB *pathway [27] and frequent *p53 *abnormalities [3,21,28]. Mutations in this gene have been reported in up to 82% of patients, compared to only 13% in the luminal A group [3].

## Relationship with BRCA-related Cancers

Patients with germline mutations in the *BRCA *genes are at risk of developing breast, ovarian, pancreatic, and prostate cancers, among other malignancies. The products of the *BRCA *genes have a variety of roles, including those relating to DNA-repair mechanisms. Cells that lack a functional *BRCA1 *or *BRCA2 *have a deficiency in the repair of DNA double-strand breaks, which is probably one of the mechanisms behind their association with increased cancer predisposition [29]. There are interesting and relevant similarities between cancers that arise in carriers of *BRCA *gene mutations and basal-like breast cancer that have led to the hypothesis that they share defects of the BRCA or related pathways. When breast cancer arises in patients with *BRCA *mutations, the majority are triple negative [30], and of the basal-like subtype in 80-90% of the cases [2,31-33]. BRCA1-related cancers similar to basal-like breast cancers tend to be characterized by a high frequency of *p53 *mutations [3,34] and genomic instability [26,32].

Mutations in the *BRCA *genes are found to be rare in sporadic breast cancers [35,36], however, recent studies have suggested that alteration in the expression or function of these or related DNA-pathway repair genes is important in the development of sporadic breast cancer [32]. Methylation of the *BRCA1 *promoter, which leads to a reduced expression of *BRCA1*, has been reported to be present in 11 to 14% of sporadic breast cancers [37,38], where it is associated with a higher histological grade and a triple-negative phenotype [37-39]. In basal-like breast cancer, the overexpression of *ID4*, a negative regulator of *BRCA1*, appears to also play a significant role in the deregulation of *BRCA1 *[40], but further studies are needed to confirm these findings. Other genes associated with *BRCA1 *in DNA repair by homologous recombination, such as *RAD51*, Fanconi's anemia proteins, CHEK2 and ATM, have also been found to be implicated in breast carcinogenesis. Whether alterations in these genes also have a role in the development of basal-like breast cancer is currently unknown and poses an interesting question for further study.

## Patients' Characteristics and Prognosis

TNBC and basal-like cancers are associated with a younger age at presentation, having a mean age of 53 years old, compared to 58 years old for other subgroups in one study [20]. Race also appears to be a risk factor, as it is more frequent in premenopausal patients of African-American heritage [19,41]. Patients with these subtypes generally present at a similar stage compared to other tumors [19], but appear to have an inferior outcome [42,43]. This inferior prognosis has been found to be independent of several other factors such as tumor grade, size and nodal status [44].

Basal-like cancers are characterized by a distinct pattern of metastasis with a predilection to metastasize to brain and lungs and less incidence of metastases to bone, liver and non-regional lymph nodes [45]. Patients with basal-like breast cancer appear have a higher incidence of locoregional failures after initial surgical treatment when compared with Luminal type A patients [46]. Interestingly, in the study by Voduc and colleagues which used IHC to determine subtype, those cancers that were triple-negative and negative for the expression of *EGFR *and CK5/6 (non-basal triple-negatives), had a lower incidence of locoregional relapse when compared to the basal-like subtype [46].

## Therapy

As stated above, there is no currently accepted specific molecular targeted agent against TNBC; however, they do appear to be responsive to chemotherapy [47]. Post-hoc analysis of several studies with diverse chemotherapy agents have shown that it is TNBC patients who seem to benefit the most from cytotoxic agents in the adjuvant setting [48-50]. Similarly, when neoadjuvant chemotherapy is administered, patients with TNBC and HER2 amplification have better response rates, as well as more frequent incidence of a pathological complete response (pCR) [51-53]; as high as 45% in a study that used 5-fluorouracil (5-FU), doxorubicin and cyclophosphamide [51]. Unfortunately, this does not translate into a better overall survival, mostly because those patients who did not achieve a complete response (CR) tend to relapse sooner than patients with other breast cancer subtypes.

There is no preferred agent in the neoadjuvant setting, although more data are definitely needed related to whether anthracycline/taxane based therapies should remain the standard approach [63].

### Platinum Agents

A group of agents particularly interesting for management of patients with TNBC are the platinum compounds, partially based on their ability to bind directly to DNA. This causes the DNA to crosslink, resulting in double-strand DNA breakage[54,55]. It has been theorized and shown in preclinical models, that neoplastic cells harboring *BRCA *mutations, and thus lacking one of the mechanisms to repair damaged DNA, are consequently more susceptible to agents that induce DNA damage [56]. A very small retrospective study that included women with *BRCA *mutations who received neo-adjuvant treatment demonstrated that patients who received cisplatin had a higher degree of pCR (83% vs. less than 22% with other regimens, not including platins) [57]. Although these data are intriguing, they should be taken with caution as the study only had 12 patients in the cisplatin cohort and it was retrospective.

In the neoadjuvant setting, single agent cisplatin was evaluated in 28 patients with TNBC which led to a pCR in six (22%) women[58]. This same group of investigators conducted a separate neoadjuvant study, this time adding bevacizumab to cisplatin. Preliminary results indicated that this combination led to a pCR in 15% (7 out of 46 patients) [59]. These results are somewhat disappointing, as the proportions of complete responses are significantly less than that achieved with multiagent neoadjuvant chemotherapy (30 - 45% in other studies) [51,52]. Because of the biochemical similarities between BRCA related breast cancers and TNBC, it has been hypothesized that TNBCs are also specifically sensitive to platinum agents. This remains a controversial topic, as to date there is no randomized, controlled study that has demonstrated the benefit of platinum versus other agents.

Cisplatin has also been coupled with other cytotoxic agents for neoadjuvant treatment; when used with epirubicin and 5-FU a pCR of 40% was achieved [60]. In a similar study of 74 patients treated with cisplatin, epirubicin and paclitaxel with G-CSF support, a remarkably high rate of pCR (65%) was seen [61]. These are encouraging results that merit further validation and testing. At the current time, however, platinum agents in the neoadjuvant setting cannot be recommended over established regimens outside of a clinical trial. Two current neoadjuvant randomized studies should help clarify the role of platinum agents in the these situations, CALGB40603 (described below in the bevacizumab section), and a Spanish Breast Cancer Research Group study [62]. In both of these trials, patients will be randomized to receive carboplatin as part of their preoperative treatment, in the Spanish study patients will receive epirubicin and cyclophosphamide for 4 cycles and then be randomized to receive docetaxel or carboplatin (NCT00432172).

In patients with metastatic disease, two clinical trials will help clarify the role of platinum agents. First, the Phase II Translational Breast Cancer Research Consortium 009 trial (NCT00483223) is evaluating the response rate of metastatic breast cancer patients treated with cisplatin or carboplatin. This trial will also evaluate, prospectively, the expression of *p63/p73 *as a potential biomarker of platinum sensitivity. These proteins are part of the *p53 *family. They are expressed in approximately one-third of patients with TNBC, and their co-expression in breast cancer cell lines results in 10-fold to 100-fold greater sensitivity to platinum chemotherapy [63]. The second study is a phase III trial currently underway in the UK, which will randomize 400 women with TNBC to carboplatin or docetaxel with crossover at progression (NCT00532727).

### Anti-tubulin Agents

A new agent that has recently been added to the armamentarium of drugs available for the treatment of breast cancer is ixabepilone. Similar to taxanes, ixabepilone stabilizes microtubules and causes cell cycle arrest and apoptosis [64]. It has the advantage of bypassing the resistance mechanisms associated with drug efflux pumps and specific paclitaxel resistance associated with *β-tubulin *[64]. Its use has been studied as a single agent in four distinct clinical trials that included 288 patients, of whom 113 (39%) had TNBC. Two phase III clinical trials have also compared ixabepilone coupled with capecitabine versus capecitabine alone. A subset analysis of women with TNBC identified an improved overall response for this combination of 31% versus 15% and a progression free survival (PFS) of 4.2 months versus 1.7 months (HR 0.63; 95% CI 0.52-0.77) [65]. In the neoadjuvant setting, treatment with ixabepilone led to a pCR in 26% of the 42 women with TNBC [66]. A retrospective analysis of this study analyzed the expression of *βIII-tubulin*, a *β-tubulin*, whose expression is correlated with resistance to taxanes. Patients with a basal-like phenotype had a higher expression of *βIII-tubulin*, and its expression was predictive of response to therapy in the overall population (area under the curve of 0.66) [67]. Further studies of the potential role of this as a predictive marker are needed before conclusions can be reached.

Another novel mitotic inhibitor currently being studied for the treatment of breast cancer is eribulin. A recently reported phase III trial compared eribulin against several investigator-chosen regimens for the treatment of women with refractory metastatic breast cancer. An improved survival in favor of those women taken eribulin was demonstrated (Median OS was 13.1 vs. 10.7, HR 0.81; 95% CI 0.66-0.99) [68]. Of the patients enrolled in this trial, 20% had TNBC. The subset analysis for this trial has not been yet reported.

### Targeted Therapies

Poly(ADP)ribose polymerase 1 (*PARP1*) is a nuclear protein that is recruited to the site of damage after the induction of both single and double stranded DNA breaks. *PARP1 *catalyzes the transfer of ADP-ribose polymers from NAD+ to target proteins, which in turn modulate DNA restoration by activating and recruiting important components of base excision repair pathway, such as XRCC1 [69,70]. *PARP1 *also contributes to the modification of histones, which leads to local chromatin remodeling, allowing access of DNA repair proteins to the repair site [71]. The inhibition of *PARP1 *potentiates the effects of ionizing radiation, DNA methylating agents, topoisomerase I inhibitors, and platinum compounds [70,72]. When *PARP1 *is inhibited in normal cells, DNA repair is done through the homologous-recombination pathway, a process for which *BRCA *is a key factor [73]. Cells that are deficient in *BRCA *are more dependent on *PARP1 *to maintain genomic integrity. Its inhibition thus leads to synthetic lethality [32,74,75], a process that occurs when inactivation of either of the two genes individually has no effect but combining the mutations is deadly to the cell [69,73]. Several *PARP1 *inhibitors are at different stages of clinical development, olaparib (previously known as AZD2281) has been evaluated in a phase 1 study where 60 patients with breast cancer were enrolled, of these, nine patients had an objective response. In addition, all the responders had abnormalities in one of the *BRCA *genes. Of the women with breast cancer, three had a *BRCA2 *mutation. A complete response that lasted in excess of 60 weeks also occurred in one of the BRCA carriers and another one had stable disease for 7 months. Olaparib was further evaluated in a phase II study that enrolled 54 patients with known *BRCA *mutations and breast cancer. The first 27 women enrolled received 400 mg twice per day, of which 11 (41%) experienced a response with a median PFS of 5.7 months. A second cohort of 27 women received 100 mg of olaparib twice per day (the lowest dose that showed an active pharmacodynamic profile in the phase 1 study). In this group, 6 patients (22%) experienced a response with a median PFS of 3.8 months. This agent was fairly well tolerated, with nausea and fatigue being the most common adverse events[76]. A recent phase I study reported by Dent et al. at the 2010 American Society of Clinical Oncology (ASCO) meeting demonstrated that it was not feasible to administer the 200 mg daily dose of olaparib in combination with weekly paclitaxel due to significant myelosuppression, in spite of prophylaxis with growth factor support [77]. Several clinical trials using olaparib in women with BRCA deficient cancers are in different stages of development (see table 1)

**Table 1:** Active Clinical Trials with the 3 most developed *PARP1 *inhibitors.

Number	Population studied	Description
Olaparib		
NCT01116648	Metastatic TNBC	Phase I/II study to evaluate optimal drug dose and establish activity of cediranib and olaparib.
NCT01078662	BRCA associated breast cancer	Phase II in patients with BRCA related cancers
NCT01115829	Metastatic TNBC	Phase 1 of the combination of cediranib plus olaparib followed by a randomized phase 2 that evaluates cediranib with or without olaparib.
NCT00516724	Metastatic TNBC	Multi-arm Phase I study that evaluates the safety and efficacy of the combination of olaparib with carboplatin, olaparib with paclitaxel or olaparib with carboplatin and paclitaxel.
NCT00647062	Metastatic TNBC or BRCA associated breast cancer	Phase 1 of the combination of olaparib and carboplatin
Veliparib		
NCT01104259	Metastatic TNBC or BRCA associated cancer	Phase I study of the combination of veliparib, vinorelbin and cisplatin
NCT01149083	BRCA associated breast cancer	Randomized phase 2 evaluating veliparib with or without carboplatin.
NCT01042379	Neoadjuvant TNBC	Multi-arm study that evaluates several regimens, One arm contains the combination of paclitaxel, carboplatin and veliparib
Iniparib		
NCT00813956	Neoadjuvant TNBC	Phase 2 study of the combination of gemcitabine, carboplatin and iniparib.
NCT01204125	Neoadjuvant TNBC	Randomized phase 2 study of paclitaxel with or without iniparib
NCT01173497	TNBC with brain metastasis	Phase 2 study of iniparib and irinotecan in women with CNS metastasis.

The similarities described above between the breast cancers that arise in patients with *BRCA *mutations and basal-like cancer have led to the hypothesis that a deficiency in a component of the *BRCA *pathway plays an important role in basal-like cancers, thus inhibition of *PARP1 *could also be an important therapeutic strategy. In a phase 2 study, 120 patients were randomized (1:1) to gemcitabine and carboplatin alone or the same combination plus the intravenous *PARP1 *inhibitor, iniparib (BSI-201). Gemcitabine (1000 mg/m^2^; IV) and carboplatin (AUC = 2; IV) were given on days 1 and 8, and iniparib (5.6 mg/kg; IV) on days 1, 4, 8, and 11 every 21 days. The addition of iniparib led to an improved response rate, (16% vs. 48%; p = 0.002) as well as PFS (3.3 vs. 6.9 months; p < 0.0001) and overall survival (12.2 vs. 7.7 months; p = 0.005; HR = 0.5; 95% CI, 0.30 - 0.82)[78-80]. The addition of iniparib was well tolerated, with no evidence of neither incremental nor new adverse effects compared to the standard arm. A confirmatory phase III clinical trial using the same regimen has completed accrual in February 2010, with data expected in 2011(NCT00938652). Iniparib is also being evaluated in 2 neoadjuvant clinical trials, NCT00813956 is a single arm trial that is studying the combination of iniparib, carboplatin and gemcitabine. The other one is a Spanish study in which patients will be randomize to received either iniparib plus paclitaxel versus placlitaxel alone (NCT01204125).

Veliparib (ABT-888) is another *PARP1 *inhibitor being evaluated in breast cancer. A recently reported study where it was used with temozolamide enrolled 41 women with metastatic disease, of which 23 (56%) had TNBC. The dose of veliparib was reduced from 40 mg to 30 mg BID due to thrombocytopenia encountered during the first cycle. In this study the activity of this combination was limited to those women who were deficient for *BRCA1 *(1 partial response (PR)) and *BRCA2 *(1 CR and 1 PR). Stable disease lasting more than 4 months was seen in 4 patients, 2 of who had a *BRCA2 *mutation. Median PFS was 1.9 months in all patients and 5.5 months in those with *BRCA *mutations[81].

It is intriguing why patients treated with oral PARP1 inhibitors had increased toxicity when these agents were used with cytotoxic chemotherapy when in contrast those patients treated with iniparib, an IV PARP1 inhibitor, had no increase toxicity.

Of note is that several studies suggest that *PARP1 *inhibitors may also be beneficial in other subtypes of breast cancer beyond TNBC. Analysis of *PARP1 *expression via IHC was done in tissue microarrays from core biopsies of 582 patients recruited to the phase III taxane-anthracycline neoadjuvant, GeparTrio trial. *PARP1 *expression was found to be present in 20% of patients with hormone receptor positive tumors, 34.4% of hormone receptor negative and HER2 positive tumors and 34.2% of TNBC. A high *PARP1 *expression was associated with higher incidence of pCR (26.5%) in patients in with high *PARP1 *expression compared to 19.1% and 8.9% in patients with medium or low expression respectively (p < 0.0005) [82]. Another clue that *PARP1 *inhibition might be beneficial in other breast cancer subtypes relates to its relationship with phosphatase and tensin homolog (*PTEN), a *phosphatase that contributes to the regulation of cell cycle progression, cell proliferation and DNA repair. Cell lines deficient in *PTEN *have an impaired homologous DNA recombination and increased cytotoxicity with *PARP1 *inhibition both in vitro and in vivo An estimated 50% of breast cancers, irrespective of their triple-receptor negativity, have a mutation in, or loss of, at least one copy of the *PTEN *gene [83,84]. Lastly, deregulation of DNA repair mechanisms and genomic instability is not exclusive of triple-negative or basal-like breast cancers, and is also commonly present in Luminal B and HER2 amplified tumors [85]. Whether using a *PARP1 *inhibitor will lead to synthetic lethality in other breast cancer subtypes is an intriguing question that is worth exploring.

The use of *PARP1 *inhibitors is at its infancy and many questions remain, such as the following: Which patients are most likely to benefit from this therapy? Are there any biomarkers that predict response to *PARP1 *inhibition besides *BRCA *mutations? What are the best cytotoxic agents to use with *PARP1 *inhibitors? What are the mechanisms of resistance to these therapies? Should *PARP1 *inhibitors be continued upon progression of the disease when introducing another cytotoxic agent? To answer such questions, new translational clinical trials are being designed and conducted.

### Other Targeted Agents

Some studies suggest that TNBC expresses *EGFR *in nearly half of the cases [9,21]. Its expression is found to be associated with an inferior outcome. A phase II study randomized patients to receive either cetuximab, an *EGFR *monoclonal antibody, alone followed by carboplatin upon progression versus concomitant cetuximab and carboplatin. Cetuximab by itself has little activity as a single agent with only 2 of 31 patients achieving a PR. When used in combination with carboplatin, it led to a PR in 13 patients and overall clinical benefit (CR+PR+ Standard disease for more than 6 months) in 19 of the 71 patients enrolled [86]. In a separate randomized phase II study, the addition of cetuximab to irinotecan and carboplatin increased RR from 30% to 49% [87]. Samples from patients enrolled in both of these trials are being studied to identify biomarkers that correlate with response to this agent [88]. A fully humanized antibody against *EGFR*, panitumumab [89], is currently being evaluated in combination with gemcitabine and carboplatin in TNBC (NCT00894504). Another approach to inhibit *EGFR *receptor signaling is with the use of small molecules that inhibit the tyrosine kinase domain of this receptor. Erlotinib, an agent of this kind, is currently being evaluated in combination with docetaxel and carboplatin in patients with metastatic TNBC (NCT00491816).

The *SRC *tyrosine kinase is a non-receptor signaling kinase that functions downstream of several growth factor receptors including *PDGFR*, *EGFR*, *IGF-1R*, and *HGFR*. It plays an important role in cancer cell proliferation and invasion through multiple pathways [90,91]. *SRC *has been found to be deregulated in breast cancer [92,93] making it a potentially important therapeutic target. Using gene expression profiling of breast cancer cell lines, two groups independently identified a gene expression pattern that was predictive of sensitivity to dasatinib, a mutitargeted thyrosine kinase that targets important oncogenic pathways, including the *SRC *family kinases [94,95]. This gene signature was present more commonly in both cell lines and in patients who had a triple-negative profile. However, dasatinib has now been studied as a single agent in TNBC with disappointing results, with only two out of 43 patients achieving a PR [96]. A currently ongoing study (NCT00780676) is evaluating whether a gene expression pattern, if present, can predict a response to dasatinib as a single agent in different subsets of breast cancers [97].

### Anti-angiogenic Agents

Angiogenesis is required for tumor growth, invasion and metastasis in several malignancies, including breast cancer. This process can be targeted with therapeutic purposes through several mechanisms. The vascular endothelial growth factor (*VEGF*) is a key mediator of angiogenesis. Its intratumoral expression has been found to be markedly elevated in patients with TNBC, compared to other subtypes [98]. Bevacizumab, a humanized monoclonal antibody against *VEGF-A*, has proven to be a valuable agent in metastatic breast cancer in several phase III clinical trials. In the E2100 study that evaluated this agent along with paclitaxel, patients who were randomized to the bevacizumab arm had an improved overall response rate of 48% versus 33% in those who received paclitaxel alone. The median PFS was significantly longer in those who received bevacizumab (11.4 versus 5.8 months, hazard ratio, 0.42; P < 0.0001), but the overall survival rate was similar in both groups (median, 26.7 vs. 25.2 months; hazard ratio, 0.88; p = 0.16)[99]. TNBC was present in 233 of the 763 (31%) patients enrolled in the E2100 trial. In this group, the PFS was increased to 10.2 months compared to 4.7 months in the paclitaxel alone arm (HR = 0.45; 95%, CI, 0.33-0.61) [100]. The AVADO trial evaluated docetaxel alone or with two different doses of bevacizumab (7.5 and 15 mg/kg every 3 weeks). Compared to placebo, PFS was superior in both bevacizumab arms, the 15 mg/kg arm was more favorable than the 7.5 mg/kg arm (median 10.0 months (15 mg/kg), HR = 0.67; P = 0.0002 and 9.0 months (7.5 mg/kg), HR = 0.80; P = 0.0450 versus 8.1 months in the docetaxel alone arm) [101]. There were 167 women with TNBC (22%), in this subgroup the addition of bevacizumab at 15 mg/kg led to an improvement in PFS from 6.0 to 8.1 months (HR = 0.60, 95%; CI, 0.39-0.92) [100]. This occurred even though the design of this study did not take full advantage of the interaction of chemotherapy plus bevacizumab, as the docetaxel was only used for a pre-set number of cycles per patient.

The RIBBON-1 trial proved that bevacizumab increased PFS and overall response rate when compared to placebo when this agent was used with single agent taxanes, anthracycline-based regimes, and capecitabine [102]. A subset analysis of patients with TNBC demonstrated an improvement in PFS when bevacizumab was used both with capecitabine (6.1 vs. 4.2 months, HR = 0.72, 95% CI, 0.49-1.06). This was also found in the taxane/anthracycline cohort (8.2 to 14.5 months, HR = 0.78, 95% CI, 0.53-1.15) [100]. A recently reported meta analysis of these 3 trials showed, as expected, a PFS advantage for patients on bevacizumab (HR = 0.64, 95% CI, 0.58-0.71)[103]. This was also true in a subset analysis of patients with TNBC (HR = 0.63, 95% CI, 0.52-0.76). However, no survival advantage was seen in the whole population or in those with triple-negative disease, which may be partially explained by the fact that there was a 60% crossover to adding bevacizumab for patients who developed tumor progression after receiving chemotherapy plus placebo. Moreover, it is important to document that this meta-analysis did demonstrate a statistically significant improvement in one-year survival for patients assigned to chemotherapy and bevacizumab versus chemotherapy and placebo. Bevacizumab is currently being evaluated in TNBC by several independent studies. CALGB 40603 (NCT00861705) is a phase II neoadjuvant study in which patients will undergo two randomizations in order to receive paclitaxel with or without carboplatin and this combination with or without bevacizumab. The second study, BEATRICE (NCT00528567) is a phase III adjuvant study where several chemotherapy regimens and different doses of bevacizumab are being evaluated in patients with TNBC. This trial recently completed accrual and the results are eagerly awaited.

### Other Antiangiogenic and Multikinase Inhibitors

Another multikinase inhibitor with antiangiogenic properties, sunitinib, has been evaluated as a single agent in a phase II study, where it was found to induce a response in 11% of a heavily pretreated cohort of metastatic breast cancer patients [104]. Unfortunately, two phase III studies have now shown that combining sunitinib with docetaxel or capecitabine does not offer any benefit in prolonging PFS compared to the cytotoxic regimen alone in patients with advanced breast cancer [105,106]. This agent is currently being evaluated in addition to carboplatin and paclitaxel as adjuvant treatment for TNBC (NCT00887575).

The mammalian target of rapamycin (mTOR) is a protein that is downstream of the PI3K/AKT pathway and, when activated, promotes protein synthesis and angiogenesis [107]. Everolimus, an mTOR inhibitor, has a 12% overall RR when used as a single agent in heavily pretreated patients with metastatic breast cancer [108]. It is currently being evaluated as a single agent in a phase II clinical trial in patients with metastatic TNBC (NCT00827567), and in a placebo controlled neoadjuvant randomized phase II study along with cisplatin and paclitaxel in patients with stages II and III TNBC (NCT00930930).

### Therapy Based on the Androgen Receptor

In an effort to further study the heterogeneity of TNBC, Doane and colleagues [109] conducted a genome wide gene expression profiling study of 99 patients with breast cancer, 41 of whom had triple negative disease. They noticed that nine of the patients with TNBC clustered together with the ER positive group. When focusing on only those patients with TNBC, the nine ER-discordant samples closely correlated with each other and were contained in a single cluster with only one additional case. Further characterization of this subtype of TNBC showed that it had a molecular resemblance to ER positive tumors and expressed genes that are targets of the ER. Half of the tumors in this group expressed the androgen receptor. Subsequently, these investigators identified MDA-MB-453 as a cell line that had a molecular phenotype similar to the previously described subtype of TNBC. This cell line, as expected, did not respond to estrogen administration but in contrast had a proliferative effect with androgen stimulation in an ER-independent but AR-dependent manner. Several studies have established that between 10-35% of TNBC express the androgen receptor [110-112]. These, and other, preclinical data have given support to the development of a phase II trial using bicalutamide, an antiandrogen, in the treatment of TNBC that are androgen receptor positive (NCT00468715).

### Other Targets

New studies that utilize high throughput technologies to assess gene expression and genomic copy number variations have provided insight into the heterogeneity of TNBC and have successfully identified potential new targets [113]. Among the targets is the fibroblast growth receptor (*FGFR*), which is part of an important signaling pathway found to be deregulated in several malignancies [114]. *FGFR1 *is overexpressed in up to 5.5% of patients with TNBC [115]. The *FGFR2 *gene has alleles that have been associated with risk of developing postmenopausal breast cancer [116]. This gene has also been found to be overexpressed in 5% of patients with TNBC[114]. Several tyrosine kinase inhibitors that target the *FGFR *receptor are currently in different stages of development [114]. One of these agents, TKI258, is currently being evaluated in a phase II study of women with HER2 negative breast cancer (NCT00958971).

Another potential target is the RAS-mitogen activated protein kinase (MAPK) signaling pathway, as it plays a central role in regulating the growth and survival of neoplastic cells. The inhibition of this pathway has been a sought after target in cancer drug development for several years. Several inhibitors of the mitogen-activated protein kinase (*MEK*), an essential component of this pathway, are in clinical trials for multiple malignancies including breast cancer [117]. Preclinical studies have demonstrated that the inhibition of *MEK *leads to the activation of the phosphatidylinositol 3-kinase (*PI3K*) pathway, a pathway that is also found to be deregulated in 30% of patients with basal-like breast cancer [84,118]. This feedback counteracts the effects of *MEK *inhibition on cell cycle and apoptosis induction [119,120]. Dual blockade, with inhibitors of both *PI3K *and *MEK*, synergistically inhibits growth of basal-like breast cancer cells *in vitro *and *in vivo *[119,120]. This combination needs to be evaluated in women with TNBC.

Finally, Speers and colleagues have used transcriptional profiling data to evaluate the expression of the human kinome. They were able to identify a set of kinases differentially expressed and critical for the growth of ER negative breast cancer [121]. In this study, two groups of TNBC were identified, a subset defined by kinases involved in cell cycle checkpoint control and mitogenesis such as *CHK1*, *BUB1*, *TTK*, and *AK2 *and another subset defined by kinases involved in the S6 kinase-signaling pathway, which includes the *RPS6KA3*, *SMG-1*, and *RPS6KA1 *kinases. The authors performed siRNA knockdown experiments to downregulate the expression of several of the kinases of interest and established that of the 20 kinases evaluated, 14 were critical for the growth of ER-negative breast cancer cell lines. The majority of these kinases are "druggable" targets that could be potentially used for therapeutic purposes.

## Conclusion

TNBC, of which the majority of cases belong to the basal-cell like phenotype of breast cancer, is a heterogeneous group. Although very likely to change in the near future, at this time, we still recommend the combination of doxorubicin plus cyclophosphamide followed by paclitaxel for patients with TNBC, in the adjuvant setting. For patients with metastatic disease, there is no standard first line agent to recommend, although the results of the ongoing phase III trial of iniparib may change the recommended standard of care, therapy should be individualized for each patient and enrollment into clinical trials is strongly encouraged. Established agents such as platinums, ixabepilone, and the antiangiogenic monoclonal antibody bevacizumab are under evaluation in both the adjuvant and the metastatic setting. The result of studies using new agents, such as inhibitors of *PARP1*, tyrosine kinases, and mTOR are currently in different phases of development and will hopefully change the paradigm of how we treat patients affected with TNBC. As new discoveries are being made, current clinical trials have translational components that we expect will provide biomarkers useful to effectively discriminate patients into those who are more likely to respond to certain therapies. The use of newer molecular techniques have and will continue to be very valuable in indentifying potential new molecules important for survival of neoplastic cells and that could potentially be targeted in the treatment of women with TNBC.

## List of abbreviations

ASCO: American Society of Clinical Oncology; CK: Cytokeratin; EGFR: epidermal growth factor receptor; ER: Estrogen Receptors; 5-FU: 5-fluorouracil; FISH: fluorescence in situ hybridization; HER2: human epidermal growth factor receptor 2; IHC: Immunohistochemistry; PgR: Progesterone Receptors; TNBC: Triple-negative Breast Cancer; CR: complete response; PR: partial response.

## Competing interests

Dr. Rafael Santana-Davila has no competing interest. Dr. Edith A. Perez has research funding from Genentech, Sanofi-Aventis, and Roche.

## Authors' contributions

RSD and EAP both made substantial contributions to conception and design, have both been involved in the drafting of the manuscript and both have given final approval of the version to be published.
